# Assessing whether the association between rheumatoid arthritis and schizophrenia is bidirectional: A nationwide population-based cohort study

**DOI:** 10.1038/s41598-018-38149-3

**Published:** 2019-03-14

**Authors:** Shih-Fen Chen, Ling-Yi Wang, Jen-Huai Chiang, Chung-Y Hsu, Yu-Chih Shen

**Affiliations:** 1 Center of Medical Genetics, Tzu Chi General Hospital, Hualien, Taiwan; 2 Epidemiology and Biostatistics Consulting Center, Department of Medical Research, and Department of Pharmacy, Tzu Chi General Hospital, Hualien, Taiwan; 3 Management Office for Health Data, China Medical University Hospital, and College of Medicine, China Medical University, Taichung, Taiwan; 4 Graduate Institute of Clinical Medical Science, China Medical University, Taichung, Taiwan; 5 Department of Psychiatry, Tzu Chi General Hospital, and School of Medicine, Tzu Chi University, Hualien, Taiwan

## Abstract

Since many studies have shown a reduction in the incidence of rheumatoid arthritis (RA) in patients with schizophrenia (SCZ), little effort has been devoted to studying this link in the Asian population. Moreover, the relationship between these two disorders could be bidirectional, but the influence of RA on the SCZ incidence is unclear. The study aims to determine whether there is a bidirectional association between RA and SCZ in an Asian population. We analyzed a 10-year population- based longitudinal cohort using the National Health Insurance Research Database of Taiwan. In the first analysis, we included a total of 58,847 SCZ patients and 235,382 non-SCZ controls, and in the second analysis, a total of 30,487 RA patients and 121,833 non-RA controls, both matched by gender, age, and index date. Cox regression analyses were performed to examine the risk of RA incidence in the first analysis and the risk of SCZ incidence in the second analysis. The main finding of this study was the discovery of a lower incidence of RA in patients with SCZ (hazard ratio (HR): 0.48, 95% confidence interval (95% CI): 0.31–0.77) after adjustment for baseline demographics and comorbidities. Additionally, the presence of RA predicted a reduced incidence rate for SCZ, but the estimate was not statistically significant (HR: 0.77, 95% CI: 0.44–1.37). The study found a unidirectional association between RA and SCZ. However, RA has an age of onset later than RA, and the protective effect of RA on SCZ incidence would be biased due to the limited number of cases.

## Introduction

Rheumatoid arthritis (RA) is a joint disorder that causes inflammation of the small joints of the hand and feet with painful, swollen and eventually eroded and fused joints^1^. Schizophrenia (SCZ) is a psychiatric disorder characterized by delusions, hallucinations, disorganized speech, disorganized behavior, and negative symptoms^2^. RA and SCZ share an impressive number of similarities. They are both chronic diseases characterized by a relapsing and remitting course^1,2^. Both diseases show a similar estimated point prevalence of 0.46% and 0.6% for RA and SCZ, respectively^3,4^. Both diseases show familial patterns of aggregation with heritability estimates of 0.65 and 0.81 for RA and SCZ, respectively^5,6^. Both diseases are considered to involve multiple genetic risk factors modified by the environment^7,8^. On the other hand, there are also differences, including age at onset (25–55 years in RA vs. 16–30 years in SCZ) and male/female ratio (1: 3 for RA and 1.4: 1 for SCZ)^9^. RA and SCZ are superficially different disorders, however, a long-standing epidemiological enigma is the reduced prevalence of RA in patients with SCZ and their relatives^10,11^.

The relationship between RA and SCZ has intrigued researchers since 1936 when Nissen and Spencer reported no arthritis among 2200 hospitalized psychiatric patients^12^. In 1992, Eaton *et al*. examined 14 studies of the relationship between RA and SCZ: 12 studies reported a lower than expected RA rate in SCZ populations and 2 did not^12^. In 1999, Oken and Schulzer performed a meta-analysis of 9 studies and concluded that RA occurs in SCZ patients at a rate of only 29% of the corresponding prevalence compared to other psychiatric patients^13^. In 2015, Euesden *et al*. reviewed 10 studies and conducted a meta-analysis reporting a significant protective effect of SCZ on RA status with an odds ratio of 0.48^11^.

Many explanations have been put forward to explain the protective effect of SCZ on the status of RA. For example, it may be a contributing factor to underreporting RA in patients with severe psychiatric conditions such as SCZ, but the prevalence of RA is not reduced in patients with other psychiatric disorders^14^. Also, differences in gender and age were not considered in early studies of the RA-SCZ relationship, but recent population-based studies have taken these differences into account and still reported reduced risks of RA in SCZ patients^10,15^. Otherwise, the reduced prevalence was observed despite the high prevalence of smoking in SCZ, which is an established risk factor for RA in the general population samples^16^. Furthermore, the protective effect of SCZ on RA may be due to the consequences of antipsychotic drugs^11^. However, many studies have been reported before the widespread use of antipsychotic drugs^12^, it is doubtful that the effects of these drugs are responsible for this correlation.

Other hypotheses that proposed to explain the protective effect of SCZ on RA, including biochemical (e.g., prostaglandin synthesis, tryptophan metabolism, and imbalance in corticosteroids), immunological (e.g., T- and B-lymphocytes, serum interleukin receptor concentration, microglia, and autoimmune), infectious (e.g., Epstein-Barr virus and Toxoplasma gondii), genetic (e.g., HLA antigen and natural resistance gene), and psychosocial (e.g., lifestyles related to social class and chronic hospitalization of SCZ patients)^9,11,14,17,18^.

Since epidemiological studies have demonstrated an association between RA and SCZ, little effort has been devoted to studying this link in the Asian population. Moreover, the relationship between these two disorders might be bidirectional, but the influence of RA on the SCZ incidence is unclear. The study aims to determine whether there is a bidirectional association between RA and SCZ using the Taiwan National Health Insurance Research Database (NHIRD). Also, such associations would be explored in different gender and age groups and depending on the presence of baseline comorbidities.

## Methods

### Data source

The National Health Insurance Program of Taiwan (NHIP) was established in 1995 and provided universal coverage through a single-payer government-mandated insurance scheme to centralize the disbursement of health care financing. As the NHIP covers about 23 million residents in Taiwan, it is one of the largest and most comprehensive population databases in the world. The NHIRD is the entire insurance claims database that includes data on health care >99% of the population of Taiwan. The database contains comprehensive information on insured persons, including demographic data, dates of clinical visits, disease diagnoses and medical procedures. Diagnostic codes were based on the International Classification of Diseases, 9th Revision, Clinical Modification (ICD-9-CM). Some subset data files have been created from NHIRD for different purposes. Two subset data files of NHIRD: Longitudinal Health Insurance Database 2000 (LHID2000) and Registry for Catastrophic Illness Database (RCID) were used for this study.

#### LHID2000

LHID2000 included 1,000,000 individuals (about 4% of the Taiwanese population) randomly sampled from the NHIRD based on those insured in 2000. LHID2000 was representative of all NHIRD. There were no statistically significant differences in age, gender, and medical costs between LHID2000 patients and the original NHIRD.

#### RCID

The Taiwan NHIP has defined several categories of serious illnesses or injuries as “catastrophic illness.” Patients had to undergo a rigorous regulatory review before obtaining a Catastrophic Illness Certificate (CIC). Patients with CIC accounted for about 4% of the Taiwanese population and received free medical care during the validity of the certificate. RCID has included all patients with CIC since 2001.

### First analysis: SCZ and incident RA

#### Inclusion of patients with SCZ and non-SCZ controls

**Figure 1:**
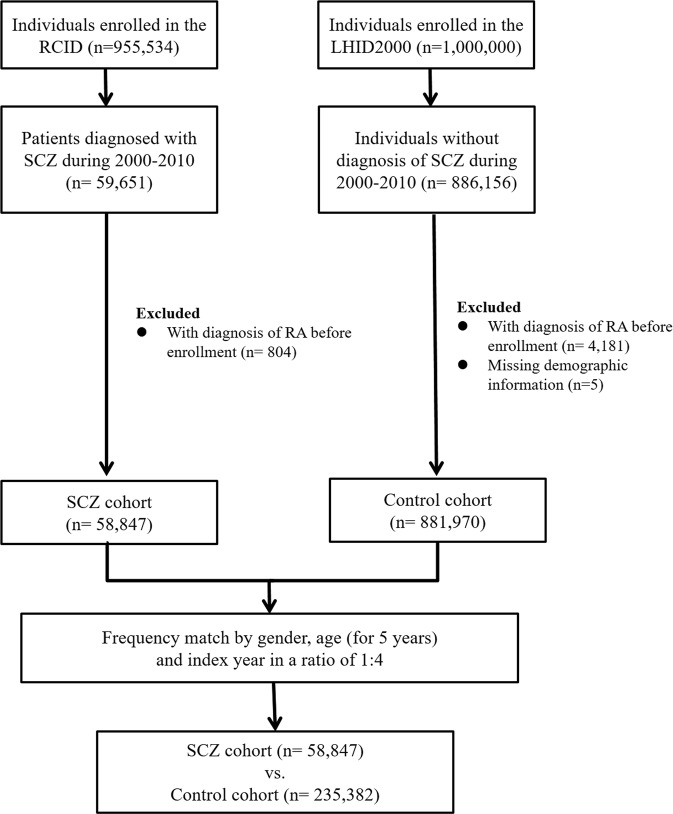
Summary diagram of the enrollment process. Abbreviations: RCID: Registry for Catastrophic Illness Database LHID2000: Longitudinal Health Insurance Database 2000 SCZ: schizophrenia RA: rheumatoid arthritis

SCZ was one of 30 categories of catastrophic diseases defined by Taiwan’s NHIP. All SCZ patients (ICD-9-CM code: 295.X) of the RCID were included in the SCZ cohort, and the first date of diagnosis was defined as the index date. Those with a history of RA between 1995 and the SCZ index date were excluded from the SCZ cohort. Four individually matched controls for each case by age, gender, and index date were randomly identified from LHID2000 after the elimination of the study cases, those who had been diagnosed with SCZ at any time (from 1995 to 2011), and those with RA between 1995 and the SCZ index date. Diagram summarizing the enrollment process was present in Figure 1.

#### Definition and incidence of RA

All patients in the first analysis were followed until the newly diagnosed RA, withdrawn from the NHIP or the end of 2011 (whichever came first). To improve the validity of the diagnosis, patients with an RA diagnosis based on the ICD-9-CM codes (714.0, 714.30–714.33) and obtained a CIC for RA were classified in incident cases.

### Second analysis: RA and incident SCZ

#### Inclusion criteria for patients with RA and non-RA controls

**Figure 2:**
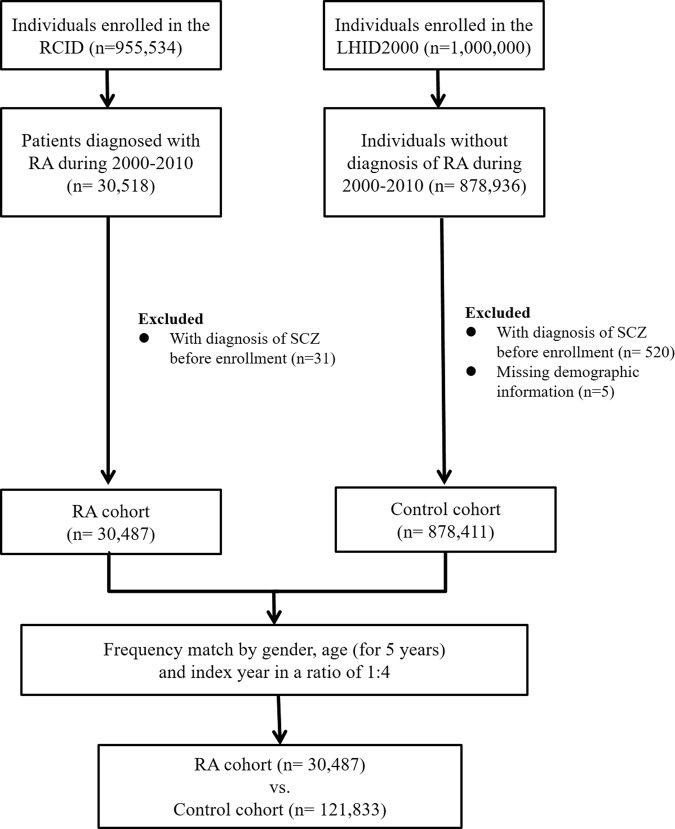
Summary diagram of the enrollment process. Abbreviations: RCID: Registry for Catastrophic Illness Database LHID2000: Longitudinal Health Insurance Database 2000 RA: rheumatoid arthritis SCZ: schizophrenia.

RA was also one of 30 categories of catastrophic diseases defined by Taiwan’s NHIP. All RA patients (ICD-9-CM code: 714.0, 714.30–714.33) of the RCID were included in the RA cohort, and the first date of diagnosis was defined as the index date. Those with a history of SCZ between 1995 and the RA index date were excluded from the RA cohort. Four individually matched controls for each case by age, gender, and index date were randomly identified from LHID2000 after the elimination of the study cases, those who had been diagnosed with RA at any time (from 1995 to 2011), and those with SCZ between 1995 and the SCZ index date. Diagram summarizing the enrollment process was present in Figure 2.

#### Definition and incidence of SCZ

All patients in the second analysis were followed until the newly diagnosed SCZ, withdrawn from the NHIP or the end of 2011 (whichever came first). Since the age of onset is generally younger for SCZ than for RA, the number of incident cases would be much lower in the second analysis than in the first analysis. In order to collect enough incident SCZ and ensure the validity of the diagnosis, we defined the incident SCZ according to the following criteria, without necessarily being serious enough to have a CIC: patients who were diagnosed with SCZ (ICD-9-CM code: 295.X) by certified psychiatrists and who received typical or atypical antipsychotics for at least 28 cumulative days (Anatomic therapeutical chemical classification codes: N05A excluding N05AN) were classified in incident cases.

### Demographic characteristics and comorbidities

Demographic characteristics of each cohort were collected, including gender, age (under 25, 25–50 and over 50), and the duration of the follow-up. We also studied baseline comorbidities in each cohort, including hypertension (ICD-9-CM: 401–405), hyperlipidemia (ICD-9-CM: 272), chronic obstructive pulmonary disease (ICD-9-CM: 491–492, 494 and 496), diabetes mellitus (ICD-9-CM: 250), asthma (ICD-9-CM: 493), chronic kidney disease (ICD-9-CM: 585), cerebrovascular disease (ICD-9-CM: 430–438), alcohol use disorder (ICD-9-CM: 303), liver cirrhosis (ICD-9-CM: 571), malignancies (ICD-9-CM: 140–239) and coronary artery disease (ICD-9-CM: 414).

### Statistical analysis

For inter-group comparisons, the t-test or Wilcoxon’s rank-sum test was used for continuous variables and the χ2 test for nominal variables, if applicable. In the first analysis, Cox regression analyses with adjustment of demographics and baseline comorbidities were performed to calculate the hazard ratio (HR) with 95% confidence interval (95% CI) of incident RA in patients with SCZ and non-SCZ controls. Sub-analyses stratified by gender and age group were also assessed for the relationship between SCZ and subsequent risk of RA. The analytical procedure in the second analysis was identical to that applied in the first analysis. In the second analysis, Cox regression analyses with adjustment of demographics and baseline comorbidities were performed to calculate the HR with 95% CI of incident SCZ in patients with RA and non-RA controls. Sub-analyses stratified by gender and age group were also assessed for the relationship between RA and subsequent risk of SCZ. The significance level of all tests was set at 0.05. We performed the full analysis by SAS 9.4 (SAS Institute Inc., Cary, NC).

### Ethics statement

This study was approved by the Institutional Review Board of China Medical University (CMUH104-REC2–115). All research methods were carried out following the relevant guidelines and regulations. Since the NHIRD only contains anonymized secondary data, the need for informed consent from individual subjects has been lifted.

## Result

### First analysis: SCZ and incident RA

#### Patient characteristics

Table 1 showed the basic characteristics of patients with SCZ and non-SCZ controls. A total of 58,847 patients with SCZ and 235,382 non-SCZ controls matched by gender and age were included in our analysis. The distribution by gender in both cohorts was predominant among male, and the average age in both cohorts was about 38 years. Most of the baseline comorbidities were statistically different between the two groups. The average years of follow-up were 7.05 and 7.73 years for the SCZ cohort and the control cohort, respectively.

**Table 1:** Demographic characteristics of patients with SCZ and non-SCZ controls.

Variable	SCZ				p-value
	No		Yes		
	n = 235382		n = 58847		
	n	%	n	%	
**Gender** ^a^					0.99
Female	110888	47.11	27723	47.11	
Male	124494	52.89	31124	52.89	
**Age at baseline, years** ^a^					0.99
<25	44086	18.73	11023	18.73	
25–50	147296	62.58	36824	62.58	
>50	44000	18.69	11000	18.69	
Mean (SD)^b^	37.84 (13.84)		37.89 (13.74)		0.48
**Comorbidities** ^**a**^					
Hypertension	27543	11.70	7011	11.91	0.15
Dyslipidemia	21521	9.14	3963	6.73	<0.01
COPD	9431	4.01	3648	6.20	<0.01
Diabetes mellitus	12961	5.51	4051	6.88	<0.01
Asthma	8440	3.59	2608	4.43	<0.01
Chronic kidney disease	1103	0.47	294	0.50	0.32
Cerebrovascular disease	7375	3.13	2620	4.45	<0.01
Alcohol use disorder	557	0.24	1739	2.96	<0.01
Liver cirrhosis	21841	9.28	6673	11.34	<0.01
Malignancies	33799	14.36	6417	10.90	<0.01
Coronary artery disease	7194	3.06	1915	3.25	0.01
**Follow-up period, years, median** ^**c**^	7.73		7.05		<0.01

^a^χ^2^ test; ^b^t-test; ^c^Wilcoxon’s rank-sum test. SCZ: schizophrenia; SD: standard deviation; COPD: chronic obstructive pulmonary disease.

#### Incidence of RA

As shown in Table 2, there were a total of 210 patients with RA during the follow-up period. The incidence rates of RA were 0.53 and 1.10 per 10,000 person-years in patients with and without SCZ, respectively. Adjusted HR for RA development was significantly lower for the SCZ cohort after controlling for other demographics and baseline comorbidities (HR: 0.48, 95% CI: 0.31–0.77). For other demographic data, the incidence of RA was higher among female than male (HR: 3.75, 95% CI: 2.66–5.27). Patients younger than 50 years had a lower incidence rate of RA than those over 50 (HR was 0.11 for patients under 25 and 0.45 for patients 25 to 50 years of age). Regarding the baseline comorbidities, none of them reached a significant difference both in the crude and adjusted model of the Cox regression analyses.

**Table 2:** Cox regression analyses of each risk factor associated with RA for the entire cohort.

Variable	RA	Person-years	IR^a^	Crude^b^			Adjusted^c^		
	n = 210			HR	(95% CI)	p-value	HR	(95% CI)	p-value
**SCZ**									
Yes	21	395134	0.53	0.48	(0.31–0.76)	< 0.01	0.48	(0.31–0.77)	< 0.01
No	189	1710857	1.10	1.00	reference		1.00	reference	
**Gender**									
Female	166	984232	1.68	4.31	(3.09–6.01)	< 0.01	3.75	(2.66–5.27)	< 0.01
Male	44	1121760	0.39	1.00	reference		1.00	reference	
**Age at baseline, years**									
<25	8	415620	0.19	0.08	(0.04–0.16)	<0.01	0.11	(0.05–0.24)	<0.01
25–50	113	1325852	0.85	0.35	(0.26–0.46)	<0.01	0.45	(0.33–0.62)	<0.01
>50	89	364520	2.44	1.00	reference		1.00	reference	
**Comorbidities**									
Hypertension	50	227314	2.20	2.61	(1.90–3.59)	<0.01	1.20	(0.81–1.78)	0.37
Dyslipidemia	39	167332	2.33	2.67	(1.89–3.79)	<0.01	1.50	(0.99–2.27)	0.05
COPD	12	82974	1.44	1.50	(0.84–2.68)	0.17	0.76	(0.40–1.42)	0.38
Diabetes mellitus	12	109738	1.09	1.12	(0.62–2.00)	0.71	0.39	(0.21–0.74)	<0.01
Asthma	14	67783	2.06	2.20	(1.28–3.78)	<0.01	1.51	(0.84–2.69)	0.16
Chronic kidney disease	1	7539	1.32	1.38	(0.19–9.81)	0.74	0.71	(0.10–5.10)	0.73
Cerebrovascular disease	15	64457	2.32	2.46	(1.46–4.17)	<0.01	1.17	(0.67–2.07)	0.57
Alcohol use disorder	3	13293	2.25	2.34	(0.75–7.32)	0.14	5.24	(1.6–17.12)	<0.01
Liver cirrhosis	28	184365	1.51	1.63	(1.09–2.42)	0.01	1.24	(0.81–1.90)	0.32
Malignancies	45	253737	1.77	2.03	(1.46–2.83)	<0.01	1.16	(0.83–1.63)	0.38
Coronary artery disease	15	56437	2.65	2.84	(1.68–4.80)	<0.01	1.20	(0.67–2.15)	0.53

^a^Incidence rates, per 10,000 person-years; ^b^Relative hazard ratio; ^c^Mutually adjusted for RA, gender, age and comorbidities in Cox regression analyses. RA: rheumatoid arthritis; SCZ: schizophrenia; IR: incidence rates; HR: hazard ratio; CI: confidence interval; COPD: chronic obstructive pulmonary disease.

#### Sub-analyses stratified by gender and age

As shown in Table 3, the two gender groups with SCZ showed the same protective association with RA, with a significant difference in female (HR: 0.48, 95% CI: 0.29–0.82) and a marginal difference in male. Also, two age groups with SCZ had a protective association with RA, with a significant difference in patients over 50 years of age (HR: 0.38, 95% CI: 0.16–0.88) and a marginal difference in those aged 25 to 50 years.

**Table 3:** Cox regression analyses of RA risk among patients with SCZ and non-SCZ controls stratified by gender and age.

Variable	SCZ						Crude^b^ HR (95% CI)	Adjusted^c^ HR (95% CI)	p-value for interaction
	No			Yes					
	RA	Person-years	IR^a^	RA	Person-years	IR^a^			
**Total**	189	1710857	1.10	21	395134	0.53	0.48 (0.31–0.76)*	0.48 (0.31–0.77)*	
**Gender**									0.99
Female	150	799635	1.87	16	184596	0.86	0.46 (0.28–0.78)*	0.48 (0.29–0.82)*	
Male	39	911222	0.42	5	210538	0.23	0.57 (0.22–1.45)	0.48 (0.18–1.30)	
**Age group, year**									0.60
<25	8	336682	0.23	0	78938	0	—	—	
25–50	98	1075116	0.91	15	250737	0.59	0.66 (0.38–1.14)	0.61 (0.34–1.07)	
>50	83	299060	2.77	6	65460	0.91	0.33 (0.15–0.76)*	0.38 (0.16–0.88)*	

^a^Per 10,000 person-years; ^b^Relative hazard ratio;: ^c^Mutually adjusted for RA, gender, age, and comorbidities in Cox regression analyses. RA: rheumatoid arthritis; SCZ: schizophrenia; IR: incidence rates; HR: hazard ratio; CI: confidence interval. *p-value < 0.05.

### Second analysis: RA and incident SCZ

#### Patient characteristics

Table 4 showed the basic characteristics of patients with RA and non-RA controls. A total of 30,487 patients with RA and 121,833 non-RA controls matched by gender and age were included in our analysis. The distribution by gender in both cohorts was predominant among female, and the average age in both cohorts was about 53 years. The majority of the baseline comorbidities were statistically different between the two groups. The average years of follow-up were 6.02 and 6.51 years for the RA cohort and the control cohort, respectively.

**Table 4:** Demographic characteristics of patients with RA and non-RA controls.

Variable	RA				p-value
	No		Yes		
	n = 121833		n = 30487		
	n	%	n	%	
**Gender** ^a^					0.94
Female	93307	76.59	23343	76.57	
Male	28526	23.41	7144	23.43	
**Age at baseline, years** ^a^					0.81
<25	5997	4.92	1528	5.01	
25–50	43520	35.72	10880	35.69	
>50	72316	59.36	18079	59.30	
Mean (SD)^b^	52.72 (15.67)		52.78 (15.67)		0.55
**Comorbidities** ^**a**^					
Hypertension	39095	32.09	10107	33.15	<0.01
Dyslipidemia	26355	21.63	5755	18.88	<0.01
COPD	12375	10.16	4548	14.92	<0.01
Diabetes mellitus	18175	14.92	4415	14.48	0.05
Asthma	8576	7.04	3270	10.73	<0.01
Chronic kidney disease	1804	1.48	624	2.05	<0.01
Cerebrovascular disease	12466	10.23	2900	9.51	<0.01
Alcohol use disorder	235	0.19	92	0.30	<0.01
Liver cirrhosis	16784	13.78	6150	20.17	<0.01
Malignancies	28547	23.43	8567	28.10	<0.01
Coronary artery disease	12600	10.34	3567	11.70	<0.01
**Follow-up period, years, median** ^**c**^	6.51		6.02		<0.01

^a^χ^2^ test; ^b^t-test; ^c^Wilcoxon’s rank-sum test. RA: rheumatoid arthritis; SD: standard deviation; COPD: chronic obstructive pulmonary disease.

#### Incidence of SCZ

As shown in Table 5, there were a total of 91 patients with RA during the follow-up period. The incidence rates of SCZ were 0.76 and 0.97 per 10,000 person-years in patients with and without RA, respectively. Adjusted HR for the development of SCZ was not significant after controlling for other demographics and baseline comorbidities (HR: 0.77, 95% CI: 0.44–1.37). For other demographic data, the incidence of SCZ was similar between female and male and between different age groups. As to baseline comorbidities, cerebrovascular disease (HR: 2.40, 95% CI: 1.35–4.29) and alcohol use disorder (HR: 22.05, 95% CI: 6.61–73.50) may be potential risk factors for SCZ incidents.

**Table 5:** Cox regression analyses of each risk factor associated with SCZ for the entire cohort.

Variable	SCZ	Person-years	IR^a^	Crude^b^			Adjusted^c^		
	n = 91			HR	(95% CI)	p-value	HR	(95% CI)	p-value
**RA**									
Yes	14	183614	0.76	0.79	(0.45–1.39)	0.40	0.77	(0.44–1.37)	0.38
No	77	793424	0.97	1.00	reference		1.00	reference	
**Gender**									
Female	74	757683	0.97	1.25	(0.74–2.12)	0.40	1.30	(0.75–2.25)	0.34
Male	17	219355	0.77	1.00	reference		1.00	reference	
**Age at baseline, years**									
<25	1	53910	0.18	0.19	(0.03–1.38)	0.10	0.31	(0.04–2.28)	0.24
25–50	37	371733	0.99	1.03	(0.68–1.57)	0.89	1.38	(0.84–2.25)	0.20
>50	53	551394	0.96	1.00	reference		1.00	reference	
**Comorbidities**									
Hypertension	36	292266	1.23	1.55	(1.02–2.36)	0.04	1.22	(0.72–2.08)	0.46
Dyslipidemia	23	187444	1.22	1.44	(0.9–2.32)	0.12	1.07	(0.61–1.88)	0.80
COPD	14	93720	1.49	1.74	(0.98–3.08)	0.05	1.44	(0.76–2.75)	0.26
Diabetes mellitus	19	130160	1.46	1.74	(1.05–2.88)	0.03	1.42	(0.79–2.55)	0.24
Asthma	8	65574	1.22	1.36	(0.66–2.82)	0.40	1.04	(0.48–2.29)	0.91
Chronic kidney disease	0	11226	0	—	—	—	—	—	—
Cerebrovascular disease	19	87018	2.18	2.73	(1.65–4.53)	<0.01	2.40	(1.35–4.29)	<0.01
Alcohol use disorder	3	1541	19.46	22.44	(7.09–71.0)	<0.01	22.05	(6.61–73.5)	<0.01
Liver cirrhosis	12	131941	0.91	0.98	(0.54–1.81)	0.96	0.70	(0.36–1.33)	0.27
Malignancies	26	209583	1.24	1.49	(0.95–2.36)	0.08	1.36	(0.86–2.17)	0.19
Coronary artery disease	10	89060	1.12	1.25	(0.65–2.41)	0.50	0.79	(0.38–1.63)	0.51

^a^Per 10,000 person-years; ^b^Relative hazard ratio; ^c^Mutually adjusted for SCZ, gender, age, and comorbidities in Cox regression analyses. SCZ: schizophrenia; RA: rheumatoid arthritis; IR: incidence rates; HR: hazard ratio; CI: confidence interval; COPD: chronic obstructive pulmonary disease.

#### Sub-analyses stratified by gender and age

As shown in Table 6, there was no significant association between RA and incident SCZ in subgroup analyses stratified by gender and age.

**Table 6:** Cox regression analyses of SCZ risk among patients with RA and non-RA controls stratified by gender and age.

Variable	RA						Crude^b^ HR (95% CI)	Adjusted^c^ HR (95% CI)	p-value for interaction
	No			Yes					
	SCZ	Person-years	IR^a^	SCZ	Person-years	IR^a^			
**Total**	77	793424	0.97	14	183614	0.76	0.79 (0.45–1.39)	0.77 (0.44–1.37)	
**Gender**									0.09
Female	65	614213	1.05	9	143470	0.62	0.59 (0.30–1.19)	0.60 (0.30–1.20)	
Male	12	179211	0.67	5	40144	1.24	1.86 (0.66–5.29)	1.66 (0.57–4.83)	
**Age group, year**									0.65
<25	0	43738	0	1	10173	0.98	—	—	
25–50	32	301233	1.06	5	70500	0.70	0.66 (0.26–1.69)	0.60 (0.23–1.56)	
>50	45	448453	1.00	8	102942	0.77	0.79 (0.37–1.67)	0.78 (0.36–1.66)	

^a^Per 10,000 person-years; ^b^Relative hazard ratio; ^c^Mutually adjusted for SCZ, gender, age, and comorbidities in Cox regression analyses. SCZ: schizophrenia; RA: rheumatoid arthritis; IR: incidence rates; HR: hazard ratio; CI: confidence interval.

## Discussion

This cohort study applies a large nationwide claims-based data to address bidirectional relationships between RA and SCZ, enabling a more powerful validation of the long-standing epidemiological enigma that has reduced the incidence of RA in patients with SCZ and testing whether the reverse association is also true. The main finding of this study was the discovery of a lower incidence of subsequent RA in patients with SCZ. On the other hand, the presence of RA predicted a lower incidence rate for SCZ, but the estimate was not statistically significant.

The finding of a lower incidence of subsequent RA in patients with SCZ is consistent with previous research and adds to the growing body of literature on this topic for the value of the same phenomenon is also found in the Asian population^11–13^. A possible hypothesis might be worth considering this finding. Both RA and SCZ have been associated with some risk alleles with genome-wide significance and negative genetic correlations^11^, suggesting that there may be shared pathogenesis at or downstream of the DNA. Some of the risk alleles may even have pleiotropic effects, that is, one allele confers a risk of SCZ, while another variant of the same allele modulates the risk of RA. In 2017, Malavia *et al*. analyzed two large databases with genome-wide significantly associated with RA or SCZ and identified 18 SNPs in 8 genes located only in the extended HLA region^19^. Genes harboring seemingly pleiotropic SNPs are closely linked to RA and SCZ associated genes through common interaction partners. Analysis of the proteins that interact with these 8 genes found more than 25 signaling pathways with proteins common to RA and SCZ signaling. Many of these pathways were associated with immune system function. The results are encouraging as they support associations of the HLA region and immune function with RA and SCZ that were known for decades.

Concerning the risk of developing SCZ as a result of RA, this is the first cohort study that applies a large national database to address this problem in the literature. This study found the presence of RA predicted a lower incidence rate for SCZ, but the estimate was not statistically significant. However, it is important to note that this conclusion must be interpreted with care. We considered this result could be partially explained in light of their respective ages at onset. SCZ has an age of onset around the age of 16–30, whereas RA has a much later age of onset around 25–55 years of age^9^. We considered that, at the age of onset of RA, the incidence rate of SCZ was low in RA and control cohorts, the protective effect of RA on SCZ incidence would be biased to zero. Also, an iatrogenic effect may also be responsible for the negative association observed in the result of RA on SCZ incidence. RA might also have a protective effect on the SCZ incidence, but RA would be treated with medications such as steroids that could increase the risk of psychosis^20^. Taken together, the effect of RA on SCZ incidence would also be biased to zero. Thus, the association between RA and SCZ incidence must be studied further.

This study found that female and older adults were potential risk factors for contracting RA, which was similar to the previous survey (2002–2007) in Taiwan^21^. In that survey, the incidence among female was about four times higher than among male. Also, the incidence of RA was low among 20–29 years old and then gradually increased to a peak in 60–69 years old. Furthermore, this study found that cerebrovascular disease and alcohol use disorder were potential risk factors for contracting SCZ. These associations can be explained in part by an immune dysfunction^22^. Evidence has indicated that chronic inflammatory processes in the comorbidities mentioned above, such as the pathophysiology of RA, involve cytokine interactions, and that this combined and increased chronic inflammatory effect can then induce SCZ^22^. Future studies are warranted to address the detail mechanisms.

This study aims to investigate whether there is a bidirectional association between RA and SCZ. A large gender- and age-matched population-based cohort with many adjusted potential risk factors are the strengths of our study. However, there are several limitations inherent to the use of claims databases that must be considered. First, to improve diagnostic validity, the diagnosis of RA and SCZ was based on the issuance of a CIC defined by the Taiwanese NHIP, which may underestimate their incidence. Second, the age of onset differs between RA and SCZ, which may bias bidirectional association analysis as mentioned above. Third, the causal relationship was assessed primarily by the chronological order in which RA and SCZ were diagnosed. A latency period may occur between the acquisition or onset of symptoms and the diagnosis of RA and SCZ, which could affect the results of observational studies such as ours. Finally, information was not available on several demographic variables such as smoking, education, lifestyle, and family history, which could have provided useful information about the factors potentially associated with RA and SCZ.

In conclusion, the study found a unidirectional association between RA and SCZ, while SCZ could predict a lower RA incidence, but RA could not predict the SCZ incidence. However, at the age of onset of RA, the incidence rate of SCZ was low, the protective effect of RA on the SCZ incidence would be biased due to the limited number of cases. Thus, the association between the RA and SCZ incidence must be studied further.
